# Genotyping validates photo‐identification by the head scale pattern in a large population of the European adder (*Vipera berus*)

**DOI:** 10.1002/ece3.3917

**Published:** 2018-02-14

**Authors:** Dirk Bauwens, Katja Claus, Joachim Mergeay

**Affiliations:** ^1^ Department of Biology Laboratory of Functional Morphology University of Antwerp Wilrijk Belgium; ^2^ Research Institute for Nature and Forest (INBO) Gaverstraat 4 9500 Geraardsbergen Belgium

**Keywords:** capture‐recapture, double‐marking, genotyping, individual recognition, natural markings

## Abstract

Capture‐mark‐recapture procedures are a basic tool in population studies and require that individual animals are correctly identified throughout their lifetime. A method that has become more and more popular uses photographic records of natural markings, such as pigmentation pattern and scalation configuration. As with any other marking tool, the validity of the photographic identification technique should be evaluated thoroughly. Here, we report on a large‐scale double‐marking study in which European adders (*Vipera berus*) were identified by both microsatellite genetic markers and by the pattern of head scalation. Samples that were successfully genotyped for all nine loci yielded 624 unique genotypes, which matched on a one‐to‐one basis with the individual assignments based on the head scalation pattern. Thus, adders considered as different individuals by their genotypes were also identified as different individuals by their head scalation pattern, and vice versa. Overall, variation in the numbers, shape, and arrangement of the head scales enabled us to distinguish among 3200+ photographed individual snakes. Adders that were repeatedly sequenced genetically over intervals of 2–3 years showed no indication whatsoever for a change in the head scale pattern. Photographic records of 900+ adders that were recaptured over periods of up to 12 years showed a very detailed and precise match of the head scale characteristics. These natural marks are thus robust over time and do not change during an individual's lifetime. With very low frequency (0.3%), we detected small changes in scalation that were readily discernible and could be attributed to physical injury or infection. Our study provides a conclusive validation for the use of photo‐identification by head scale patterns in the European adder.

## 1 INTRODUCTION

Studies of the ecology of populations provide basic information for understanding the dynamics of population numbers and are fundamental in applied fields such a conservation biology, wildlife management, and pest control. Population studies often rely on capture‐mark‐recapture procedures to derive traits of populations from the characteristics of the composing individuals (Southwood & Henderson, 2000). This requires animals to be individually recognizable and distinguishable from their conspecifics. To achieve this, a variety of marking techniques has been developed (reviewed in Silvy, Lopez, & Peterson, 2012).

Over the past 2–3 decades, natural markings have been increasingly used as an identification tool (Speed, Meekan, & Bradshaw, 2007). This procedure uses photographic images of distinctive natural features, such as color pattern to identify individual animals at capture and recapture occasions. Photographs are stored in a (digital) library to facilitate cross‐matching of the images. Potential matches of the individually unique natural marks can be detected and evaluated by eye or by photo‐identification software (e.g., Van Tienhoven, Den Hartog, & Reijns, 2007; Sacchi et al., 2010; Bolger, Morrison, Vance, Lee, & Farid, 2012; Hartog & Reijns, 2014; Moya et al., 2015). Photographic identification by natural markings is especially appropriate when live animals are difficult to capture (e.g., large marine and terrestrial mammals), or when capture and handling cause severe stress to the study organisms. As with any other marking method, the validity of the photographic identification technique depends on the fulfillment of two basic assumptions. First, all marked animals are correctly identified upon each capture, requiring that the natural markings are sufficiently variable and distinctive to allow individual recognition. Second, markings must be permanent, that is, the natural marks are robust over time and do not change during an individual's lifetime.

**Figure 1:**
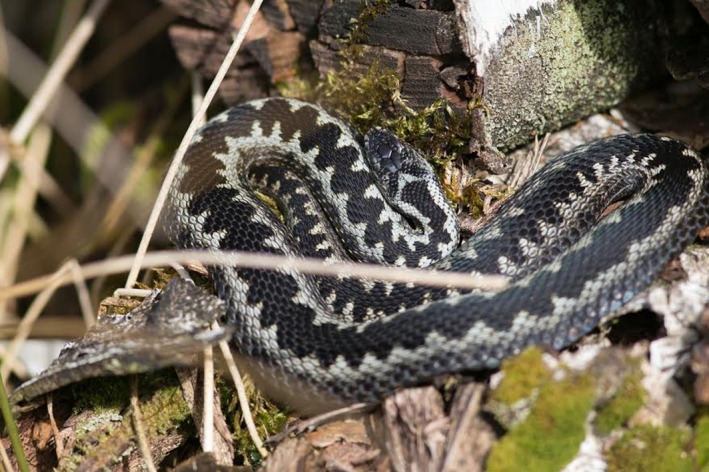
An adult male of the European adder (*Vipera berus*) that has recently shed its skin. Individual snakes can be identified by their head scalation pattern (photograph by Rudi Segers)

The European adder (*Vipera berus*) (Figure 1) features enormous variation in its ground coloration and in head and body markings (Biella, 1990; Völkl & Thiesmeier, 2002). This makes it a good candidate for photographic identification. Accordingly, Sheldon and Bradley (1989) developed a simple procedure to identify and code individual adders on the basis of images of their head markings. They also observed that individual markings remained constant over several years, at least in adult adders. An alternative method for individual recognition of adders exploits the extreme variation in the number, shape, and arrangement of the head scales (Benson, 1999; Biella, 1988; Stoyanov & Tzankov, 2017). Embryological studies of reptiles have shown that scalation traits, including skull plates, are formed during the late stages of embryogenesis (Maderson, 1965; Maderson & Alibardi, 2000) and presumably remain constant during a reptile's lifespan. In addition, numerous fieldworkers have frequently demonstrated stability in head scale patterns on the basis of recaptures of individual adders and other vipers over periods as long as 10+ years (Benson, 1999; Üveges, Halpern, Péchy, Posta, & Komlósi, 2012). However, such observations do not deny the possibility that scale pattern changes do occur but go unnoticed, so that a single individual is erroneously identified as another individual upon consecutive captures. This possibility was strengthened by Tomović, Carretero, Ajtíc, and Crnobrnja‐Isailovíc (2008) who reported frequent and profound changes in head scalation patterns in both immature and adult meadow vipers (*V. ursinii*), a sister species of *V. berus*. Such ontogenetic changes would obviously invalidate the use of scale patterns as an identification tool. However, a subsequent long‐term study of a captive population of the meadow viper found no evidence of any postnatal instability of head scales (Üveges et al., 2012). These contradictory findings urge the need for a thorough study on the reliability of head scale traits as an identification tool in vipers.

The validity of the head scale identification procedure should best be tested by a solid double‐marking system, allowing for parallel verification of an animal's identity. The use of two marking systems in the same population indeed provides two independent sources of identification and an opportunity to examine identification errors. In this study, we report on the results from a large‐scale double‐marking study in which adders were identified by both morphological and genetic markers. Data were gathered during the course of a long‐term citizen science project in a large population of the adder. Our objectives are threefold: (1) to compare individual assignments obtained by the examination of head scalation patterns and by genotyping; (2) to assess and illustrate the extent of variation in head scale configurations and explore its potential to identify large numbers of snakes; and (3) to examine stability of the scalation pattern during the snakes’ lifetime and expose the rare occasions of observed scale changes.

## 2 MATERIAL AND METHODS

### 2.1 Study area and photographic identification

This citizen science project was initiated in 2000 by K.C. as a spare‐time research activity. Over the years, she trained several volunteers who participated in the fieldwork. These efforts have led to a long‐term population study (2000–present) of adders in the “Groot Schietveld” (ca 1,570 ha; N 51°20–22′–E 4°32‐37′, province of Antwerp, Belgium). The area is used (since 1893) as a military exercise zone, and access is restricted to authorized persons and only when there are no military activities (mainly nonworking days and hours). It is a lowland area (altitude ranges 18–25 m above sea level) covered by a mosaic of heathlands, moors, fens, and woodlands.

Adders are very abundant in this area; total population size is tentatively estimated to be in the order of several thousand individuals. Snakes are found over the entire area, but we concentrated capture efforts to 11 study sites (1–8 ha each; total search area: 46.5 ha). Snakes were located by sight while walking slowly and erratically through the terrain, captured by hand and released immediately after handling. We used a compact camera to shoot a digital photograph of the upper side of the head and recorded date, time, exact location (GPS coordinates), sex, snout–vent (SVL) and tail length (to the nearest 5 mm), and body mass (to nearest 1 g). Recently shed skins found in the field were collected and photographed when head scales were well preserved.

**Figure 2:**
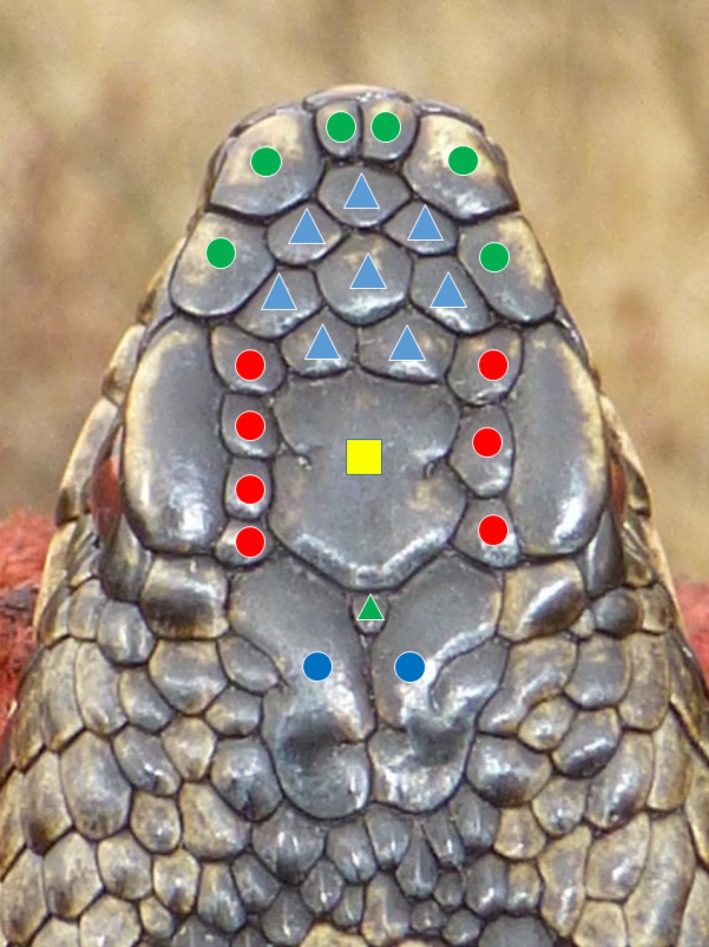
Example of the “typical” head scale pattern of the European adder showing the predefined groups of scales used to assign a score to each pattern. Scale groups were as follows: apicals + canthals (green dots), intercanthals (blue triangles), parafrontals (red dots), frontal (yellow quare), parietals (blue dots), interparietals (green triangle)

During the years 2000–2016, we collected a total of 5,986 images of the head scale pattern of 3,215 individual snakes. Each photographic image was stored in a digital collection and coupled to a database storing the information recorded at each capture. We coded each scalation pattern to facilitate visual comparison and cross‐matching of newly taken with previously stored photographs. This multicharacter score denoted the number of scales in predefined groups (Biella, 1990; Völkl & Thiesmeier, 2002) (Figure 2). When one or more head shields could not be allocated with certainty to a single group, two or more scores were assigned to that photograph. Although several snakes could obtain the same code because they exhibit a globally similar scalation pattern, usage of the code cuts down the number of images to be compared visually. In addition, specification of some basic characteristics of the adder (sex, age class, capture location) will further reduce the candidate list of photographed snakes. Finally, when a matching pattern is found in the image library, the picture is labeled with the corresponding snake's unique identification number. Otherwise, it is assigned a new identification number. This procedure does not take more than 30 s–2 min by a trained observer. All identifications were always double‐checked by two independently operating persons.

### 2.2 Genetic sampling and sequencing

In 2011–2013, we collected genetic samples for genotyping. Samples were either (1) cloacal swabs; (2) pieces of tail skin obtained from molting animals; (3) shed skins found in the field.

These samples were transported to the genetic laboratory of the Research Institute for Nature and Forest Research (INBO, Geraardsbergen, Belgium) for processing and genotyping.

Total DNA was extracted using QIAamp DNA Mini Kits (Qiagen). We amplified nine polymorphic nuclear microsatellites that were specifically developed for *Vipera berus* (Vb‐A8, Vb‐A11, Vb‐B'2, Vb‐B10, Vb‐B'10, Vb‐B18, Vb‐D'10, Vb‐3, and Vb‐D71 (Carlsson, Isaksson, Höggren, & Tegelström, 2003; Ursenbacher, Monney, & Fumagalli, 2009). PCR was performed in two 10 μl multiplex reactions, containing each 5 ng of genomic DNA, 1× Qiagen Multiplex PCR Master, and fluorescently labeled primers: 0.2 μmol/L Vb‐A8, 0.05 μmol/L Vb‐A11, and 0.1 μmol/L of the remaining primers. Reactions were performed on a Biometra T1000 thermocycler, involving 15′ at 94°C, 30 cycles of 30″ 94°C, 45″ 56°C, 45″ 72°C, and a final extension of 10′ at 72°C, followed by storage at 4°C until electrophoresis. Microsatellites markers were run on an ABI 3500 capillary DNA sequencer (Applied Biosystems) and scored using the program GeneMapper 4.1 (Applied Biosystems). To assess genotyping errors, we included two independent PCR amplifications for each of 10 samples.

We retained for further analyses 797 samples that were successfully genotyped for the complete set of nine loci. The program GENECAP 1.4 (Wilberg & Dreher, 2004), a Microsoft Excel macro, was used to match samples with identical microsatellite genotypes.

## 3 RESULTS

### 3.1 Genetic and photographic identification

The genetic analyses revealed a total of 53 alleles with an average of 5.9 alleles per locus (range: 2–10). The repeated samples provided identical fingerprints; hence, there was no evidence for amplification or scoring errors.

We identified 624 unique genotypes. The Sib and HW probabilities of identity (Wilberg & Dreher, 2004) were, respectively, 3.77E−03 and 4.802E−06. Our database included 141 genotypes that were sampled two to five times each. Most importantly, there was a one‐to‐one match of all 624 genotypes with the individual assignments that were based on the head scalation pattern. Samples that were identified as recaptures by genetic markers matched with images considered as recaptures by the photo‐identification procedure. Thus, adders considered as different individuals by their microsatellite genotype were also recognized as different individuals by their head scalation pattern, and vice versa.

The genotypes that were repeatedly sequenced included a considerable number (*n* = 74 out of 141) of which the samples were collected over a period of 2–3 years. In this group of adders, no indication whatsoever was found for a change in the head scale pattern.

### 3.2 Variation in head scalation traits

**Figure 3:**
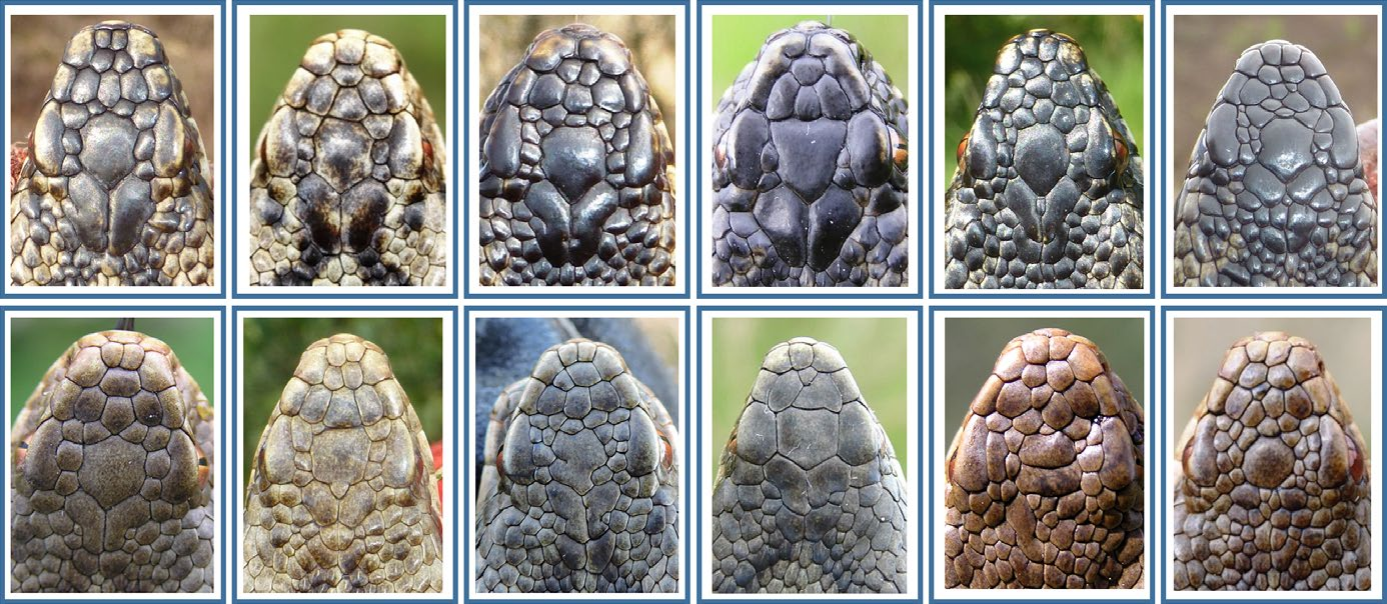
A sample of photographic images showing individual variation in head scalation pattern in adult male (top row) and adult female (bottom row) adders. We intentionally only show images of adult adders, to exclude aberrant scalation patterns that could lead to lower survival abilities during immature life stages. The two rightmost scalation patterns on the bottom row resemble those of hybrids between *Vipera berus* and *V. aspis* from western France (compare to drawings in Table 1 of (Guiller et al., 2016))

**Figure 4:**
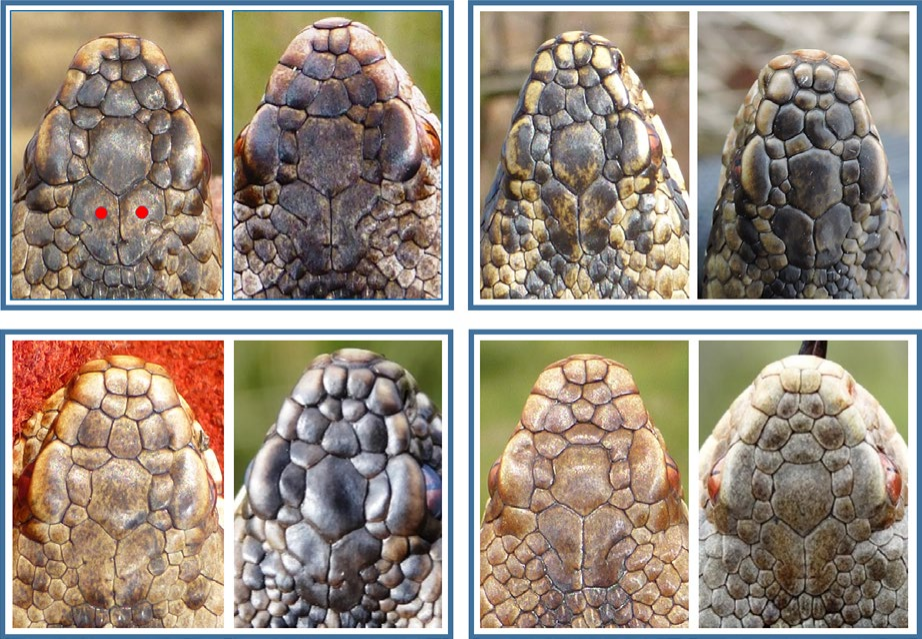
Comparison of four pairs of individual adders with highly similar patterns of the larger scales in the anterior head part. Examination of the shape of the parietal scales (marked with a red dot in the upper left image) and of the number and shape of the smaller scales that border them allows discrimination between the individuals within each pair

We observed tremendous variation in the numbers, shape, and arrangement of the head scales (Figure 3), enabling us to distinguish among 3,215 individual snakes photographed during 2000–2016. Relative to the “typical” head scalation of the adder (Figure 2), the main sources of among individual differences were the fragmentation and the (in)complete fusion of shields, variation in their shape, and differences in the arrangement of the smaller head scales (i.e., the intercanthals and parafrontals; Table 1). Less common was the fusion of scales that bordered the pileus (i.e., the apicals and canthals). Although arrangement of the scales was often quite symmetric, some notably asymmetric configurations were observed (Figure 3). In about half the individuals, the typically large and paired parietal scales were subdivided into four up to 10+ smaller fragments. Fragmentation of the frontal shield occurred less often. In the rare instances where we doubted about the individual assignment on the basis of the number and pattern of the scales in the anterior head part, examination of the shape of the parietal shields and of the smaller scales that bordered them was decisive (Figure 4).

**Table 1:** Summary statistics (first line: median (interquartile range), second line: range) of the number of head scales in distinct scale groups (see Figure 2) in male (*n* = 1,678) and female (*n* = 1,535) adders

	Parietals	Interparietals	Canthals	Intercanthals	Parafrontals left	Parafrontals right
Males	2 (2–3)	0 (0–0)	6 (6–6)	7 (5–8)	3 (3–4)	3 (3–4)
	1–10	0–5	4–6	3–13	0–8	0–8
Females	3 (2–4)	0 (0–1)	6 (6–6)	7 (6–8)	3 (3–4)	3 (3–4)
	2–15	0–7	0–6	2–14	0–8	0–8

### 3.3 Individual changes in head scalation pattern

**Figure 5:**
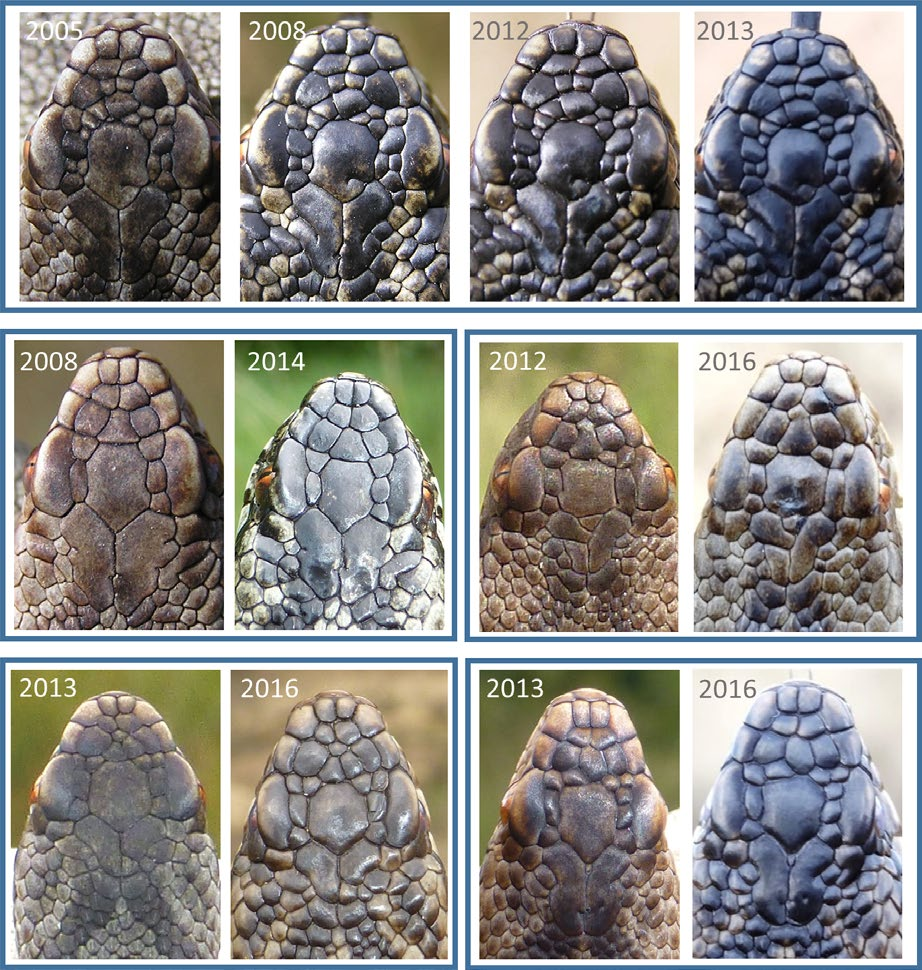
Examples of the stability of the head scalation pattern in five adders. The first image of each animal was taken within weeks after its birth, and the next images at their recapture as an adult several years later. Numbers denote the years wherein the adder was photographed

In our restricted sample (*n* = 74) of adders that were repeatedly genotyped during more than 1 year, no evidence for instability of the head scalation traits was detected. Similarly, the photographic records of 904 individuals that were recaptured after one to 12 years showed no indications for changes in scalation pattern. These included a considerable number (*n* = 62) of adders first identified in their first or second year of life and recaptured as adults, three or more years later (Figure 5). The photographic records at recapture not only reveal the stability of the overall scalation pattern, but also of the small incisions and incomplete fragmentations of the shields (Figure 5).

**Figure 6:**
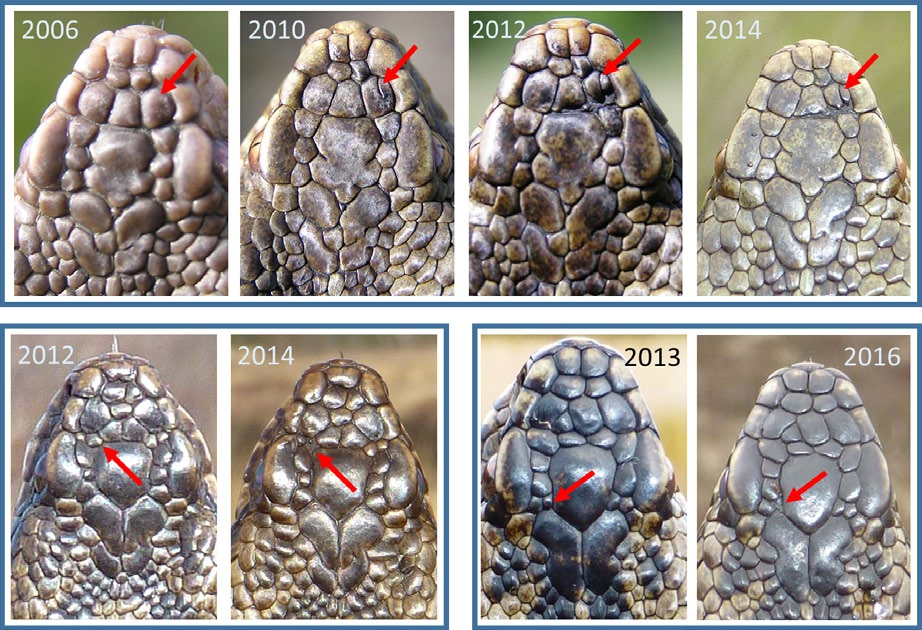
Examples of observed changes in the head scale pattern at successive captures. The adder on the top row exhibits a gradual splitting of a scale in two parts, and their progressive reduction in size. The bottom row shows the disappearance of a single small scale in two adders

In a very small number of individual adders (*n* = 9, or 0.3% of total), we observed minor changes in the scale pattern (Figure 6). These involved the gradual splitting of a scale (*n* = 4) or the disappearance of a single scale (*n* = 5). They were easily detected by the presence of a wound epithelium and the irregular shape and small size of the newly formed scales, and could be attributed to injury or disease. We stress that all observed changes only occurred with small scales.

## 4 DISCUSSION

Double‐marking studies provide a reliable way to compare the performance of two independent individual recognition procedures, but have rarely been applied to studies of natural markings (but see (Stevick, Palsbøll, Smith, Bravington, & Hammond, 2001; Gosselin, Sainte‐Marie, & Sévigny, 2007; Drechsler, Helling, & Steinfartz, 2015)). Our double‐marking study convincingly demonstrates that photographic records of the head scale pattern provide a trustworthy method for identifying individual adders. In a large sample of snake individuals (*n* = 624), there was a one‐to‐one match between the individual assignments obtained by photographic records and by genetic markers. Thus, performance of the photographic identification method equals that using genetic markers. The main difference is that the latter procedure is technically much more elaborate and therefore more expensive.

Photographic identification of adders by their scale markings provides a valid alternative to more invasive methods such as scale clipping or inserting a passive integrated transponder (PIT) tag. Being a noninvasive alternative, it has no potential negative effects on the behavior and survival of the snakes. Moreover, it is cheap and easy to apply. Although we collected most of our photographs after capturing the snake, to assure that high‐quality images were obtained, in some instances, we successfully relied on pictures taken in the field from unrestrained animals. Hence, it is no surprise that the method has been employed in (citizen) science projects (e.g., Hodges & Seabrook, 2014, http://www.herpetofauna.co.uk/forum/individual-adder-id_topic1365.html, http://www.ravon.nl/RAVONActief/Werkgroepen/AdderonderzoekNL/tabid/1656/Default.aspx). These studies are often directed toward and have implications for the conservation of populations and their habitats. Hence, it is reassuring that our results provide strong evidence for the reliability of the method. We note that we effectively recognized 3200+ individual adders in our extremely large study population. Virtually all adder populations studied to date are much smaller and rarely exceed an estimated number of 100 (adult) individuals (e.g., Prestt, 1971; Madsen et al., 2000; Ursenbacher et al., 2009). We therefore expect that photographic identification by scalation patterns can be successfully employed in all adder populations and by extension also to other species of Eurasian vipers. Regretfully, it is inappropriate for many other species of snakes, in particular Colubrids, as they lack individual variation in head scalation patterns.

The observed individual variation in the head scalation pattern was virtually limitless. Only on rare occasions did we encounter two or more patterns that looked quite similar at first sight. Careful consideration of all details then allowed to decide whether the photographed adder was an unknown or a recaptured animal (see Figure 4). Most of the individual variation reflected the fusion or fragmentation and the shape and arrangements of the smaller scales in the anterior part of the head. Most remarkable was the fragmentation of the largest elements of the pileus, that is, the frontal and especially the parietal scales. In some of our adders, fragmentation of these shields was so pronounced that they resembled individuals from western France that were shown to be natural hybrids of *V. berus* and *V. aspis* (Guiller, Lourdais, & Ursenbacher, 2016). Such hybrids are, however, unlikely to occur in our area, as the nearest population of *V. aspis* is located at 240 km SE. Instead, these scalation patterns underscore the extent of individual variation present in our population.

Photographic identification was aided by labeling the scalation pattern with a simple multicharacter code, which was based on the number of scales in predefined groups. Together with specification of whole‐animal traits (e.g., sex, age class), this considerably reduced the number of images to be compared visually. Alternatively, automatic pattern recognition by image comparison software (e.g., Bolger et al., 2012; Hartog & Reijns, 2014; Moya et al., 2015) could be used. However, application of this method to reptile scalation patterns requires the digitization of reference points on each photograph (Sacchi, Scali, Mangiacotti, Sannolo, & Zuffi, 2016; Sacchi et al., 2010). Following this labor‐intensive and time‐consuming phase, photographs in the collection are ranked by the software program according to their overall similarity with the searched image, but final visual verification is still needed. Hence, we strongly doubt whether the use of such an “automated” technique would lead to much time gain relative to our simple method.

A critical condition for all marking techniques is that marks should not be lost and should not change during the animal's lifetime. The use of the head scalation pattern to identify individual snakes thus requires that the pattern is fixed at birth and does not change during their further life. Scalation traits of reptiles become differentiated and their configuration is established during the late stages of embryogenesis (Alibardi, 1996; Maderson, 1965; Maderson & Alibardi, 2000). It is generally accepted that the number and arrangement of head shields and scales do not change during postnatal life (e.g., Dohm & Garland, 1993; King, 1997; Lourdais, Shine, Bonnet, Guillon, & Naulleau, 2004; Lorioux et al., 2013). Our study confirms this view as no indication of scalation changes was found in a considerable number (*n* = 74) of genetically marked adders that were recaptured over a period of 2–3 years. In addition, a large number of snakes that were identified by their head scalation pattern were recaptured after periods of up to 12 years. Photographic records taken at the consecutive captures showed a very detailed and precise match of the general pattern, but also of small incisions and partial subdivisions of scales. This was evident not only in adders that were first captured as adults, but also in snakes that were initially photographed as newborn and recaptured as adults, three or more years later. The very rare changes in scalation that we observed involved the presence of a wound epithelium and small neogenic scales. These alterations were readily discernible and could be attributed to physical injury or infection (Maderson, Baranowitz, & Roth, 1978). Similar cases have sporadically been reported for other adder populations (R. van Leeningen, pers. comm.).

Our results match and extend those of other studies that failed to find any or very infrequent postnatal changes in scalation patterns in *Vipera* species (e.g., Üveges et al., 2012; Hodges & Seabrook, 2014; Stoyanov & Tzankov, 2017). By contrast, Tomović et al. (2008) claimed to have observed clear‐cut changes in head scales over a period of 1–4 years in 12 of 23 (= 52%) recaptured meadow vipers. If a similar frequency had applied to our study population, we would expect to detect similar changes in at least a fraction of the genetically fingerprinted individuals that were recaptured over intervals of comparable length (i.e., 2–3 years). However, no changes were detected in any of these adders. Tomović et al. (2008) did not provide any hypothesis on the putative causes of the changes, except that they were not induced by injury or other trauma. Given that their results have not been confirmed to date by other studies, we suggest that the observed postnatal scalation shifts should be attributed to extremely rare and unknown (environmental) conditions. Alternatively and more parsimoniously, their primary marking system by scale clipping may have induced identification errors due to regeneration or damaging of scales (Plummer & Ferner, 2012).

In summary, our results provide very strong support for the ontogenetic stability of the head scale pattern in the adder and provide a conclusive validation for the use of photo‐identification by head scale patterns in adders.

## CONFLICT OF INTEREST

None declared.

## AUTHOR CONTRIBUTIONS

D.B. and K.C. conceived the study, coordinated and participated in data collection and data analyses. J.M. coordinated and supervised the genotypic laboratory analyses. All authors contributed to writing the manuscript and approved the final version.
